# Paresthesia and forearm pain after phlebotomy due to medial antebrachial cutaneous nerve injury

**DOI:** 10.1186/1749-7221-6-5

**Published:** 2011-09-06

**Authors:** Mahsa Asheghan, Amidoddin Khatibi, Mohammad Taghi Holisaz

**Affiliations:** 1 Department of Physical Medicine and Rehabilitation, Baghyatollah Hospital, Baghyatollah University of Medical Sciences, Mollasadra Street, Tehran, Iran

## Abstract

**Back ground:**

Although phlebotomy is a common procedure, there is limited information concerning to documented complications of venipuncture.

**Case presentation:**

A 45 year old left- handed woman was refered for elecrodiagnostic study with dysesthesia and pain in left medial forearm. She noted these symptoms three weeks after phelebotomy. Electrodiagnostic study showed severe involvement of left side Medial Antebrachial Cutaneous nerve (MAC nerve).

**Conclusion:**

Phelebotomy is a cause of MAC nerve injury. Electrodiagnostic testing can be helpful in evaluating cases of sensory disturbance after phlebotomy.

## Background

The MAC nerve can be damaged by a number of iatrgenic causes. Phlebotomy is a rare cause of injury.

We present a case of phlebotomy-induced injury to the MAC nerve, in which the diagnosis was made using nerve conduction study. According to our knowledge, this is the first case in which electrodiagnostic studies were used to document venipuncture-related injury of the MAC nerve. The use of electrodiagnostic test for diagnosis of this type of injury, has only been reported one time for radial nerve and three for lateral antebrachial cutaneous nerve [1-4].

## Case presentation

**Figure 1:**
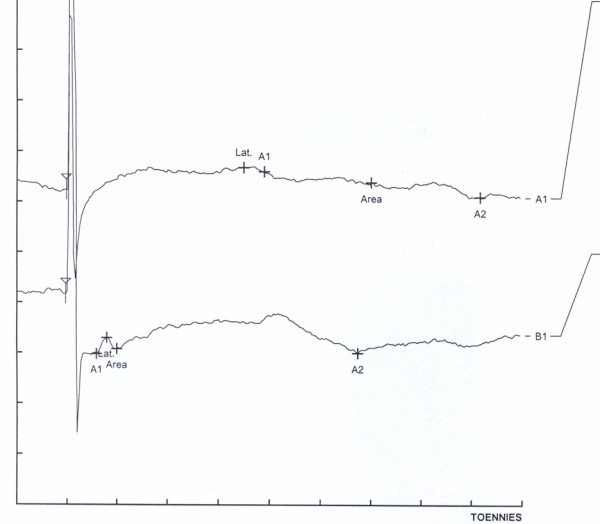
**Nerve conduction responses of both sides MAC nerve, lower trace: normal response obtained from Rt side MAC, Latency: 1.9 ms, Ampl: 18.5 μV**. Upper trace: no response obtained by left side MAC nerve stimulation.

A 45 year left-handed woman was referred to electromyography clinic for electrodiagnostic study because of hypoesthesia over the medial aspect of left forearm. She complained of a shooting pain along with the onset of dysesthesias over there. She noted this complaint since three weeks ago after routine venipuncture. The patient had no history of polyneuropathy, chronic systemic disease or surgical intervention at the elbow. Physical examination showed normal inspection and muscles power but decreased sensation of pinprick and light touch in anteromedial aspect of forearm. Electrodiagnostic study was performed on bilateral MAC nerves using routine technique [5]. The study revealed absence of sensory nerve action potential from the left MAC nerve, and normal in right side [Figure 1].

For confirmation the diagnosis, we tried several times with stimulation in various points in cubital fossa and with high stimulation current up to 45 mA. All other nerves in left upper limb were normal in nerve conduction studies. The diagnosis was severe injury to left MAC nerve after phlebotomy. Unfortunately we could not follow the patient for further evaluation.

## Discussion

The MAC nerve originates from the medial cord or rarely the lower trunk, and is derived from segments C8 and T1. This nerve is subcutaneous just proximal to the medial epicondyle and course along the medial aspect of the arm to provide cutaneous sensation to the medial forearm. Some sort of MAC nerve injury in literature includes steroid injection for medial epicondylitis, cubital tumor surgery, elbow arthroscopy, orthopedic surgery, tumor and routine venipuncture [6-12]. It is also caused more rarely by repeated minor trauma, soft tissue laceration and tuberculoid leprosy neuritis or subcutaneous lipoma [13-15]. Several reports of nerve injuries after phlebotomy included injury to lateral antebrachial cutaneous nerve, MAC, radial, dorsal ulnar sensory branch in hand [16,17]. Because of superficial position of nerves in cubital fossa, these are susceptible to injury during phlebotomy especially where multiple attempts done. This is the first case in which electrodiagnostic studies were used to make the diagnosis of a direct injury of the MAC nerve as a result of phlebotomy.

MAC nerve injury may be undiagnosed, because the nerve is purely sensory and there is no motor abnormality. On the other hand, the electrophysiological studies of MAC nerve is not a part of routine upper extremity electrodiagnostic examinations. The clinician and the electromyographer should be aware of to this condition. In conclusion, MAC nerve injury should be taken into account for the differential diagnosis of the patients with complaints of pain and dysestesia in medial forearm and anteromedial aspect of the elbow. Electrodiagnostic study should be used for evaluation of patients with complaint of neurologic symptoms after phlebotomy to diagnose the location and severity of the injury.

## Consent

Written informed consent was obtained from the patient for publication of this case report

## Competing interests

The authors declare that they have no competing interests.

## Authors' contributions

MA carried out the electrodiagnosis studies. AK participated in the design of the study. MTH conceived of the study, and participated in its design and coordination. All authors read and approved the final manuscript.
